# Chromatic spatial contrast sensitivity estimated by visual evoked cortical potential and psychophysics

**DOI:** 10.1590/1414-431X20122428

**Published:** 2013-02-01

**Authors:** M.T.S. Barboni, B.D. Gomes, G.S. Souza, A.R. Rodrigues, D.F. Ventura, L.C.L. Silveira

**Affiliations:** 1 Instituto de Ciências Biológicas, Universidade Federal do Pará, Belém, PA, Brasil; 2 Núcleo de Medicina Tropical, Universidade Federal do Pará, , Belém, PA, Brasil; 3 Instituto de Psicologia, Universidade de São Paulo, São Paulo, SP, Brasil

**Keywords:** Contrast sensitivity, Color vision, Steady-state visual evoked cortical potential, Visual thresholds, Spatial vision, Psychophysics

## Abstract

The purpose of the present study was to measure contrast sensitivity to equiluminant gratings using steady-state visual evoked cortical potential (ssVECP) and psychophysics. Six healthy volunteers were evaluated with ssVECPs and psychophysics. The visual stimuli were red-green or blue-yellow horizontal sinusoidal gratings, 5° × 5°, 34.3 cd/m^2^ mean luminance, presented at 6 Hz. Eight spatial frequencies from 0.2 to 8 cpd were used, each presented at 8 contrast levels. Contrast threshold was obtained by extrapolating second harmonic amplitude values to zero. Psychophysical contrast thresholds were measured using stimuli at 6 Hz and static presentation. Contrast sensitivity was calculated as the inverse function of the pooled cone contrast threshold. ssVECP and both psychophysical contrast sensitivity functions (CSFs) were low-pass functions for red-green gratings. For electrophysiology, the highest contrast sensitivity values were found at 0.4 cpd (1.95 ± 0.15). ssVECP CSF was similar to dynamic psychophysical CSF, while static CSF had higher values ranging from 0.4 to 6 cpd (P < 0.05, ANOVA). Blue-yellow chromatic functions showed no specific tuning shape; however, at high spatial frequencies the evoked potentials showed higher contrast sensitivity than the psychophysical methods (P < 0.05, ANOVA). Evoked potentials can be used reliably to evaluate chromatic red-green CSFs in agreement with psychophysical thresholds, mainly if the same temporal properties are applied to the stimulus. For blue-yellow CSF, correlation between electrophysiology and psychophysics was poor at high spatial frequency, possibly due to a greater effect of chromatic aberration on this kind of stimulus.

## Introduction

The visual system of mammals, including primates and humans, is organized in parallel visual pathways that originate in the retina and are distributed to several subcortical targets in the mesencephalon and diencephalon. It is understood that the visual pathways more important for visual perception of form, movement, and several spatial and temporal aspects of vision are those that connect the retina to the lateral geniculate nucleus (LGN) and primary visual cortex (V1): the M (magnocellular), P (parvocellular), and K (koniocellular) pathways, named according to the relay layers of the lateral geniculate nucleus 1,2. Shapley and Perry 1 established the original distinction and significance between M and P cells. A recent review about the morphological and physiological properties of such cells may be found elsewhere 2, providing readers with the appropriate baseline references. M cells comprise about 10% of all ganglion cells and are very sensitive to achromatic contrast but their response saturates at high contrast levels, and they do not respond to pure chromatic contrast. They project to the LGN magnocellular layers and from there cells with similar properties project to V1 layer 4Cα 1,2. P cells comprise about 80% of ganglion cells and are insensitive to low levels of achromatic contrast but their response continues to increase in amplitude when contrast is raised to high levels, and they respond to red-green contrast. They project to the LGN parvocellular layers and from there cells with similar properties project to V1 layer 4Cβ 1,2. K cells comprise a heterogeneous group of ganglion cells and LGN cells, some of them responding to blue-yellow chromatic contrast, and project to V1 layer 4A 3. In addition to the visual cortex, at least two main visual streams provide visual information to the visual and visuomotor areas located in the dorsal and ventral regions of the cerebral cortex 3.

While numerous psychophysical studies have demonstrated the spatial contrast sensitivity function (CSF) using achromatic stimuli e.g.,^4^-^7^, fewer investigations have looked at the psychophysical chromatic spatial CSF 6,8-11. This is also the case for human electrophysiological studies. The achromatic spatial CSF was measured by means of threshold estimation using noninvasive electrophysiology such as the visual evoked cortical potential and both steady-state VECP (ssVECP) and transient VECP 6,7, 12-16. Visual contrast thresholds for achromatic spatial patterns, such as gratings, have been estimated by using ssVECPs at as many as 18 spatial frequencies, providing a detailed electrophysiological account of the human achromatic spatial CSF 15. The study of chromatic spatial CSF using VECP was limited to a few studies and chromatic spatial or temporal CSFs based on electrophysiological threshold estimates are rare in the literature both for red-green temporal CSF 17 and red-green spatial CSF 6. Chromatic spatial contrast sensitivity was more often studied based on suprathreshold VECP amplitudes 18,19, and additional studies comparing electrophysiological chromatic versus achromatic CSFs, as well as comparing electrophysiological and psychophysical chromatic CSFs.

The linear correspondence between VECP and contrast has been well established by other studies either using achromatic gratings 7,12,20,21 or equiluminant chromatic gratings 21-27, as well as equiluminant sinusoidal plaid patterns 6,17. Thus, we estimated the chromatic contrast sensitivity from pooled cone contrast thresholds 28 by recording ssVECPs in response to red-green and blue-yellow gratings at a wide range of contrast levels.

## Subjects and Methods

### Subjects

Six healthy trichromats (28.3 ± 3.5 years old) with normal acuity or corrected to 20/20 participated in the study. Normal trichromacy was verified using Ishihara's pseudoisochromatic plates and a custom-made computerized version of the 100-hue Farnsworth-Munsell color arrangement test. Only the eye with lower dioptric error was tested. Inclusion criteria were absence of ophthalmologic and degenerative diseases or diseases that could affect the visual nervous system. All subjects gave written informed consent prior to the test. Tests were performed according to the tenets of the Declaration of Helsinki and were approved by the Human Research Ethics Committee, Núcleo de Medicina Tropical, Universidade Federal do Pará (Report #113/2004, November 25, 2004), according to Resolution #196/96 of the National Health Council of Brazil.

### Visual stimulation

The Visage platform (Cambridge Research System, UK) was used to generate stimuli. The stimuli were displayed on a 20″ Diamond Pro 2070 CRT monitor, 100-Hz frame rate, 800 × 600 pixels (Mitsubishi Electric, Australia). Gamma correction was performed using a ColorCAL colorimeter (Cambridge Research System). Stimulus luminance and chromaticity were measured with a CS-100A Chroma Meter (Konica Minolta, USA).

Visual stimuli consisted of horizontal red-green and blue-yellow equiluminant gratings displayed in 180° phase reversal at 6-Hz temporal frequency, i.e., 12 reversals per second, with square temporal modulation. Eight spatial frequencies from 0.2 to 8 cpd were evaluated. A central cross (1° of visual field) was used for fixation. Eight contrast levels were used for each spatial frequency. Chromaticities were at the highest contrast: red, u′ = 0.288, v′ = 0.480; green, u′ = 0.150, v′ = 0.480; blue, u′ = 0.219, v′ = 0.420; yellow = 0.219, v′ = 0.540. Stimuli were displayed against a background of the same mean luminance (34.3 cd/m^2^) and chromaticity (u′ = 0.219, v′ = 0.480). The reason to use these coordinates is that in our previous studies they were effective to elicit robust VECP amplitudes and are optimized to stimulate psychophysically color opponent pathways 25,26.

Equiluminance was achieved for each subject and spatial frequency by heterochromatic flicker photometry with temporal frequency at 20 Hz. In this procedure, for each stimulus, the subject had to diminish the flicker sensation as much as he could. Luminance from each grating color after flicker fusion was then added to each stimulus used in all tests.

We used Judd modified values (*x′*, y′, z′) 29 to obtain tristimulus values and the Smith and Pokorny 30 cone fundamentals to calculate cone contrast. As a single measurement of cone contrast, we calculated the pooled value:
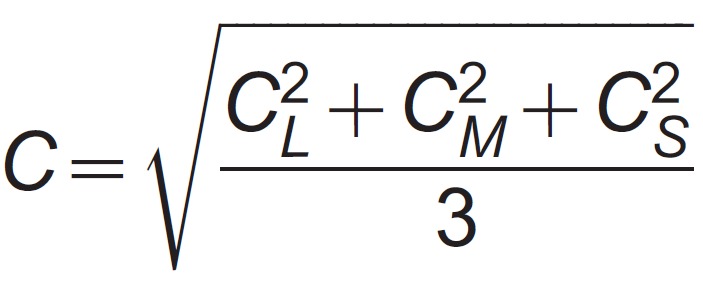
(Equation 1)where *C* is the pooled cone contrast, and *C_L_* , *C_M_* , *C_S_* are the cone contrast for the *L*, *M*, and *S* cones, respectively. The use of pooled cone contrast provides an objective measurement of a color stimulus and has been used in a variety of studies of the human color vision to represent not only chromatic stimuli but also color discrimination thresholds. In addition, cone contrasts take into account the first stage of color processing, i.e., the absorption of photons as a function of wavelength 31. The chromaticity coordinate as well as the cone contrasts for each color are shown in Table 1 for the maximum chromatic contrast.

**Table:**
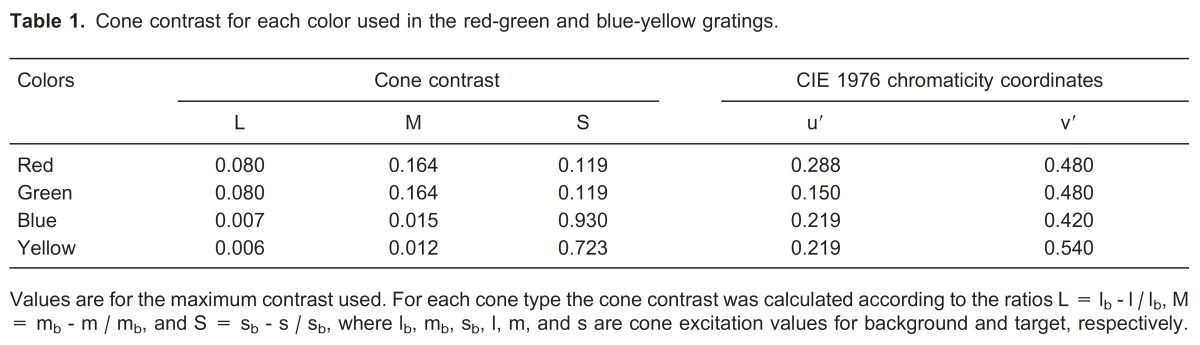

### Electrophysiological procedure

The electrophysiological procedure followed the guidelines of the International Federation of Clinical Physiology (IFCN) 32. Gold-cup electrodes were used to obtain one-channel recordings from Oz (active electrode), Fp (reference electrode), and Fpz (ground) according to the International 10/20 System. The recordings were sampled at 1 kHz and amplified 50,000× and on-line band-pass filtered between 0.3 and 100 Hz. For each condition, 120-240 epochs, 1 s each, were averaged. The evoked potential signals were amplified with a CED™ 1902 pre-amplifier and recorded with a CED™ 1401 device (Cambridge Electronic Design Ltd., UK). VECPs were analyzed after fast Fourier transform (FFT) to obtain the amplitude of the second harmonic (12 Hz) measured in the frequency domain. This amplitude was then used as a chromatic sensitivity index. To determine if signals were above noise level, the statistical significance of the ssVECP was estimated as indicated by Meigen and Bach 33. According to this method, the signal-to-noise ratio (SNR) was calculated by the relation SNR = Hamp_12_ / (Hamp_11_ + Hamp_13_ / 2), where Hamp_12_, Hamp_11_, and Hamp_13_ are the amplitudes of harmonics at the frequencies 12, 11, and 13 Hz, respectively. From some assumptions about the spectral properties and the probability density of Fourier components at the stimulus frequency, for a significance level of 5%, the critical SNR is 2.82. This means that a value above 2.82 is considered to be significantly different from noise. Contrast threshold was estimated by extrapolation of straight line functions to zero amplitude. The contrast sensitivity was calculated as the inverse function of the pooled cone contrast threshold.

### Psychophysics

In order to compare electrophysiological and behavioral data, a psychophysical procedure was implemented in two conditions: 6-Hz phase reversal, as used in the electrophysiological measurements, and static presentation. Thresholds were determined by the adjustment method. In this procedure, for each grating and spatial frequency the stimulus was first shown at the highest contrast and then the subjects had to decrease chromatic contrast until they barely saw the stimulus. The contrast was then recorded and, in the next step, the starting contrast was shown with 1.5 dB relative to the last contrast recorded. This reduced the time of the procedure and avoided anticipation error. A threshold was then assumed as an average of six independent trials at each spatial frequency.

## Results

### Electrophysiology

**Figure 1:**
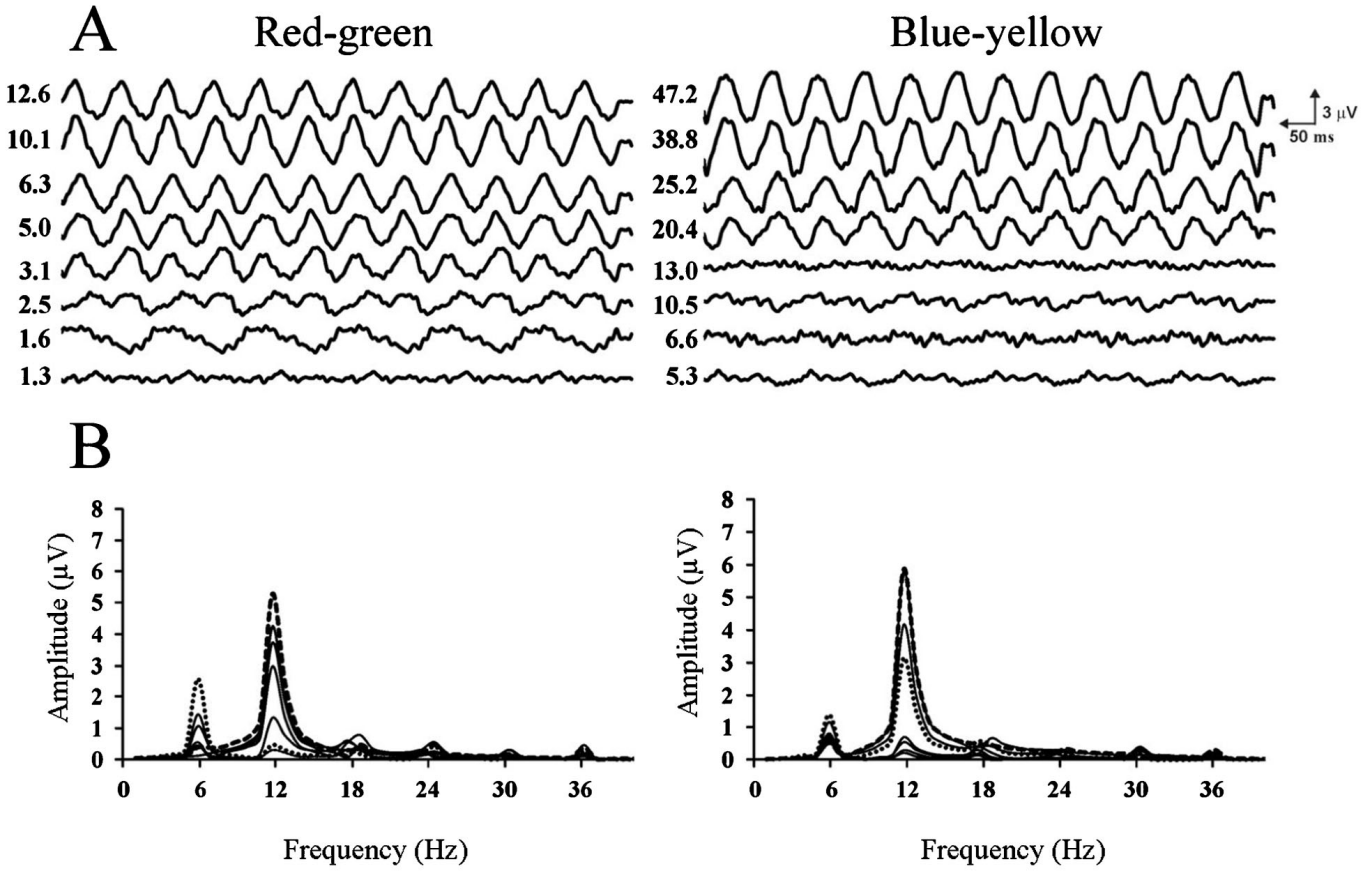
*A*, Average recordings for all subjects obtained for 2 cpd in both conditions, red-green and blue-yellow grating stimulation. The numbers on the left of each recording are pooled cone contrast values. *B*, Fast Fourier transform spectra from the recordings in *A*. Dashed lines are spectra for 80% of the maximum contrast used for both stimuli, which correspond to pooled cone contrast of 10.1 and 38.8 for red-green and blue-yellow gratings, respectively. Dotted lines are spectra for 12.5% of the maximum contrast for red-green gratings and 40% for blue-yellow gratings. They correspond to the pooled cone contrast of 1.6 for red-green and 20.4 for blue-yellow gratings.

**Figure 2:**
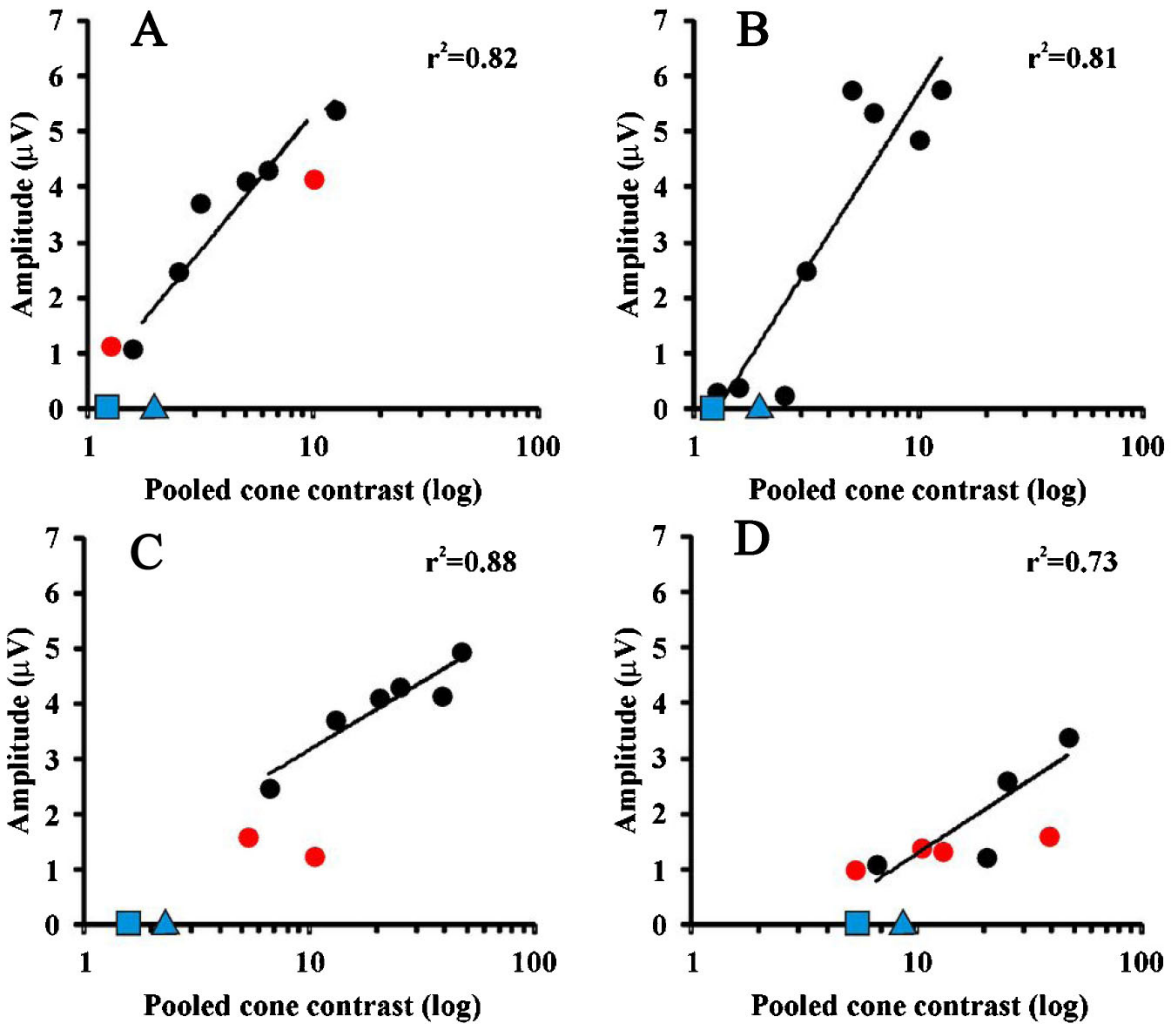
Amplitude variation plotted against pooled cone contrast for one of the subjects tested. Pearson's correlation coefficients are shown. Black filled circles were used to estimate regression lines. Red filled circles are amplitude data that were not different from noise. *A* and *B*, data for red-green gratings at 2 and 4 cpd, respectively. *C* and *D*, data for blue-yellow gratings at 2 and 4 cpd, respectively. Blue squares and blue triangles are thresholds estimated with static and 6-Hz psychophysics, respectively.

Figure 1 shows mean time-averaged ssVECP waveforms and FFT spectra for all subjects at different pooled cone contrasts for the spatial frequency of 2 cpd. Spectra showed a peak at 12 Hz corresponding to the number of reversals per cycle and an additional peak at 6 Hz more prominent at the lowest contrasts, where the response of first harmonic did not differ from noise. A comparison is shown in Figure 1 with different pooled cone contrasts for high and low responses according to the contrast. As blue-yellow stimulation elicited responses that quickly decreased with contrast in comparison to red-green stimulation, an abrupt response decrease occurred for pooled contrasts below 20.4, which corresponded to 40% of the maximum contrast used. The second harmonic amplitude as a function of log pooled cone contrast was well fitted by linear functions (Figure 2). Correlation coefficients were higher for the red-green than for the blue-yellow amplitude modulation.

**Figure 3:**
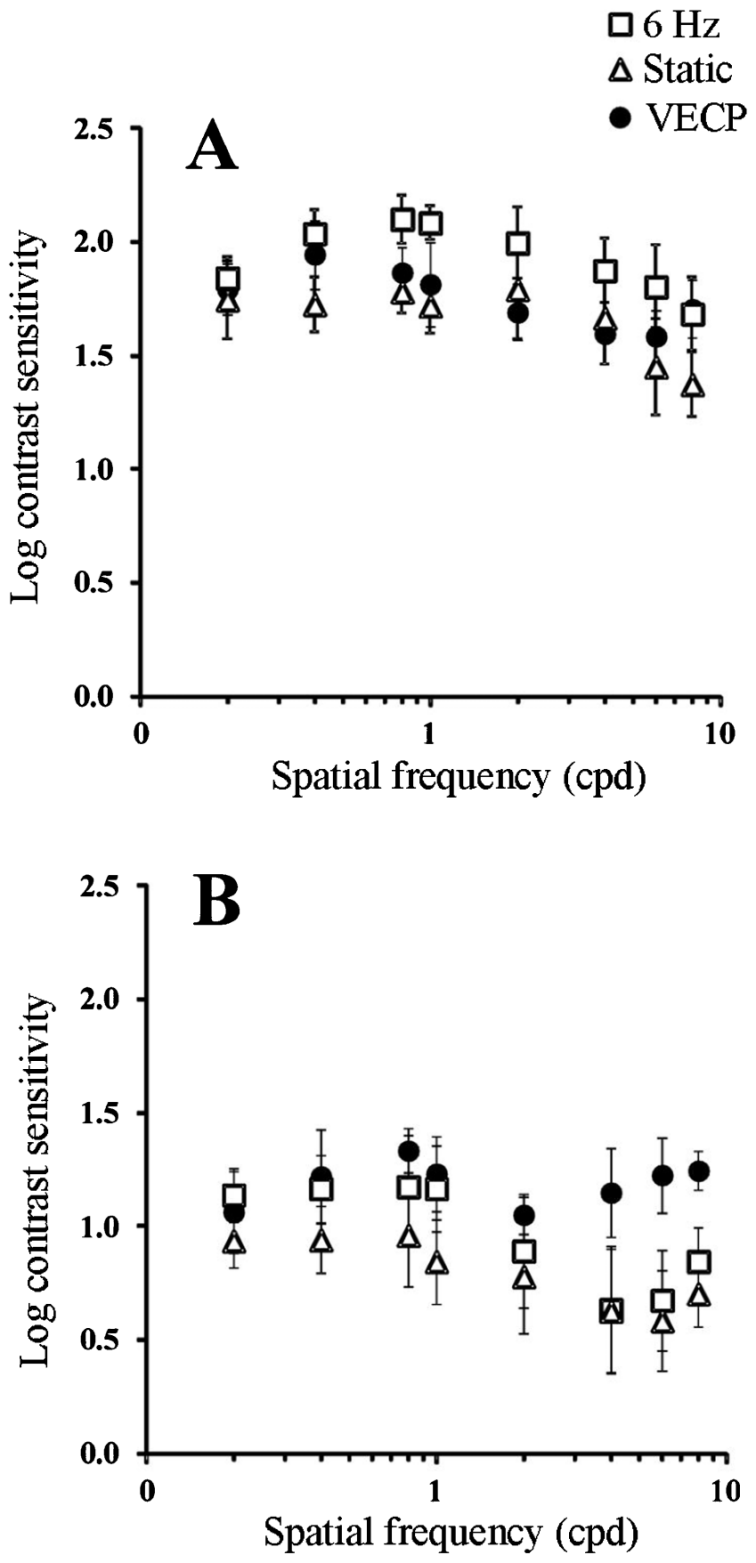
Contrast sensitivity to red-green (*A*) and blue-yellow (*B*) sinusoidal gratings obtained as the inverse of contrast thresholds estimated for 6 normal trichromat subjects. Data are reported as means ± SD for 6 subjects. Filled circles and empty triangles are contrast sensitivity data from visual evoked cortical potential (VECP) and psychophysics using the same temporal parameters as in VECP, respectively. Empty squares are contrast sensitivity data from psychophysics using the same gratings but static presentation. Contrast sensitivity values measured with ssVEP were compared with each psychophysical procedure and also a comparison between psychophysics tests was made (one-way ANOVA, α = 0.05). When using red-green gratings, ssVEP function was similar to 6-Hz psychophysics but different from static psychophysical contrast sensitivity function (one-way ANOVA, α = 0.05). For blue-yellow gratings ssVECP was different from both psychophysical conditions (one-way ANOVA, α = 0.05).

For red-green gratings, the highest contrast sensitivity values were found at 0.4 cpd (1.95 ± 0.1) and the lowest sensitivity values were observed at 4 cpd (1.60 ± 0.0) and 6 cpd (1.58 ± 0.1; Table 2, Figure 3A). For blue-yellow gratings, the highest contrast sensitivity values were found at 0.8 cpd (1.33 ± 0.1) and the lowest at 2 cpd (1.05 ± 0.1; Table 2, Figure 3B). Red-green function showed a small attenuation at low spatial frequencies and a more pronounced attenuation at high spatial frequencies. At the highest spatial frequency tested, 8 cpd, the sensitivity started to increase, an indication of luminance intrusion due to chromatic aberration. Thus, chromatic aberration did not permit us to extend the study further at higher spatial frequencies. Blue-yellow function showed a similar trend, but at 4 cpd the sensitivity started to increase, an indication that luminance intrusion was more severe for this kind of chromatic stimulus than for red-green gratings.

**Table:**
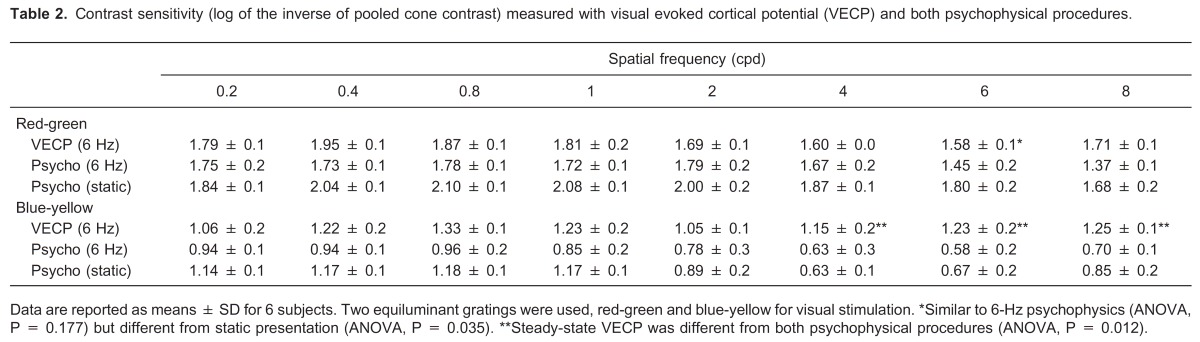

### Psychophysics

Red-green CSFs obtained with 6-Hz stimulation and static presentation showed a small attenuation at low spatial frequencies and a more accentuated attenuation at high spatial frequencies similar to the electrophysiological results for red-green gratings (Figure 3A). Blue-yellow CSFs showed a trend similar to red-green CSF up to 4 cpd, but at higher spatial frequencies (6 and 8 cpd) there were signs of luminance intrusion to chromatic aberration (Figure 3B). The static red-green CSF provided higher values than the 6-Hz red-green CSF (one-way ANOVA, α = 0.05). For blue-yellow gratings no significant difference was found between the two psychophysical conditions (one-way ANOVA, α = 0.05).

### Comparison between VECP and psychophysics CSF

For red-green gratings the electrophysiological function was similar to psychophysics 6-Hz CSF but different from static psychophysics CSF (one-way ANOVA, α = 0.05). The static psychophysics CSF was higher than ssVECP and 6-Hz psychophysics CSFs (Table 2, Figure 3A). When using blue-yellow gratings as stimuli, ssVECP was different from both psychophysical conditions (α = 0.05, one-way ANOVA). This difference was observed mainly at the higher spatial frequencies (Table 2, Figure 3B).

## Discussion

**Figure 4:**
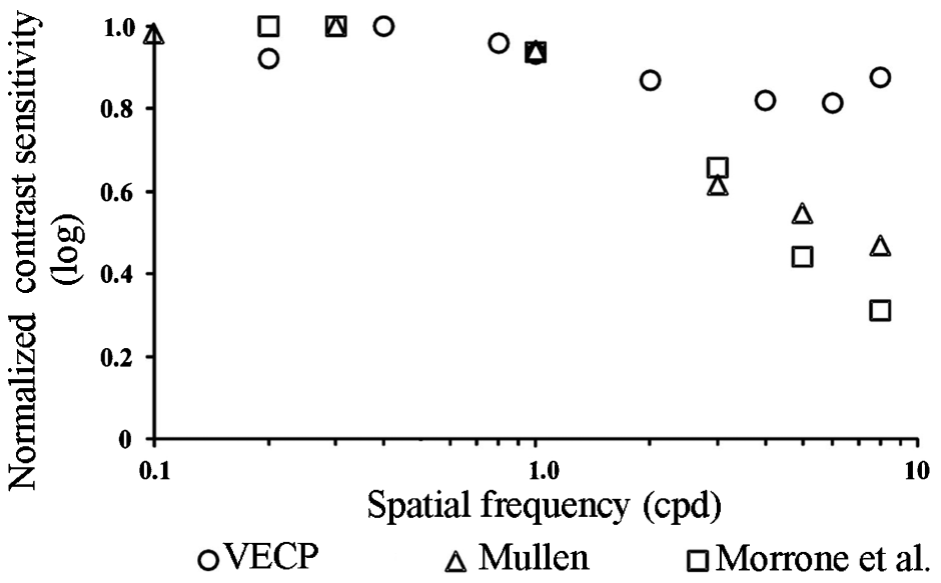
Comparison of red-green contrast sensitivity measured with evoked potentials or psychophysics in three different laboratories. Empty triangles and squares represent data reproduced from the Figure 6 of Mullen 11 and Figure 10 of Morrone et al. 6, respectively. Morrone et al. 6 used a 5-Hz sinusoidal red-green plaid pattern as a stimulus and plotted steady-state visual evoked cortical potential (ssVECP) as a function of Michelson contrast following the Mullen psychophysical study of chromatic contrast sensitivity 11. Empty circles are data from the present study, obtained by using 6-Hz sine wave gratings as a stimulus and plotting ssVECP amplitude as a function of pooled cone contrast. Due to the different conditions used in the two studies, the curves were normalized to their peak value to provide a basis for comparison. The results were generally similar up to 2-3 cpd. From 4 cpd onwards the two data sets diverged and those from the present study showed higher values than those of Mullen and Morrone et al. See text for discussion.

We were able to measure ssVECP amplitude as a function of pooled cone contrast for red-green and blue-yellow sine wave gratings. From these measurements, it was possible to estimate contrast thresholds and provide color contrast sensitivity over a range of spatial frequencies. Morrone et al. 6 measured red-green contrast sensitivity along a similar range of spatial frequencies as the one used in the present study (they did not study blue-yellow contrast sensitivity as we did in this research). Similarly to our study, they estimated chromatic contrast thresholds by extrapolating the ssVECP amplitude versus contrast fittings to the zero level. However, the methodology they used differed from that used in our study in many aspects: 5-Hz temporal frequency, sinusoidal plaid patterns as spatial stimuli, Michelson contrast as contrast metrics according to the study of Mullen 11. In contrast, in the present study we used 6-Hz temporal frequency, sine wave gratings, and pooled cone contrast metrics. In Figure 4, we compare our results with those of Morrone et al. 6 and Mullen 11. Due to the different conditions used in the three studies, data sets were normalized to their peak values. The results were generally similar up to 2-3 cpd. From 4 cpd onwards the data sets diverged and those from the present study showed higher values than those of Morrone et al. 6 and Mullen 11. A possible explanation for this difference is the intrusion of luminance artifacts due to chromatic aberration under our experimental conditions, which became significant for sine wave gratings only for medium to high spatial frequencies, while the optical procedure used by Morrone et al. 6 and Mullen 11 avoided this, at least in the range of spatial frequencies used by them.

Regan 21 analyzed steady-state evoked potentials for a variety of chromatic contrasts at a single-spatial frequency using red-green gratings and checkerboard patterns. Our results agree with Regan's findings regarding the good correlation between evoked potentials and psychophysical thresholds. In the present study, this agreement between VECP and dynamic psychophysics was extended to thresholds estimated at a range of spatial frequencies. There was also a relationship with Regan's results regarding the amplitude saturation we found mainly at the higher contrasts, as can be seen in Figure 2.

An important issue is the use of pattern reversal to assess chromatic responses. There is evidence that steady-state onset/offset stimulation using equiluminant chromatic gratings could be more effective to elicit responses that are related to the activity of color opponent pathways. We have previously and successfully used 1-Hz onset/offset stimulation to elicit responses along a variety of color axes [Bibr B25] 25,26 and also compared these responses with those from onset/offset achromatic gratings 34. When using equiluminant gratings to obtain transient instead of steady-state VECP, the opposite polarity elicited by the appearance of the chromatic and achromatic gratings might make it difficult to measure the chromatic signal 22,25,26,34-36. This difference in morphology seems consistent with non-overlapping activation of magnocellular and parvocellular pathways. However, there is no such indication of color response selectivity in steady-state VECP. McKeefry et al. 37 compared VECP elicited by gratings displayed in onset/offset and reversal at three temporal frequencies. Two of the frequencies were high enough to give rise to steady-state VECP and thus higher amplitudes in the frequency domain. They reported decreased amplitude for reversal in comparison to onset/offset in the time and frequency domain mainly at higher temporal frequencies. However, using onset/offset leads to some second harmonic contribution evident at 8.33 Hz, which indicates a magnocellular activity intrusion. The authors suggested a magnocellular origin for the harmonic measured in pattern reversal even though they used equiluminant chromatic gratings. This is not in agreement with our previous results 26, which also showed higher amplitudes for steady-state onset/offset in comparison to pattern reversal in the time and frequency domain. However, pattern reversal amplitude was more persistent at the lowest contrasts, providing lower VECP thresholds than onset/offset 26. In addition, single-cell recording showed that many neurons in V1 respond robustly to pure color and luminance stimuli. Skottun and Skoyles 38 pointed out several reasons that make it difficult to consider second harmonic response as a good measurement of magnocellular activity in chromatic ssVECP. Thus, we suggest that our results were evoked mainly by contribution from color opponent pathways.

### Evoked potentials and psychophysics for red-green gratings

Our results for red-green ssVECP at different spatial frequencies showed a low-pass function with a decrease in amplitude at the higher spatial frequencies, in agreement with studies that measured suprathreshold amplitude against contrast variation. Arakawa et al. 19 studied ssVECP suprathreshold amplitude responses at nine spatial frequencies varying from 0.5 to 8.0 cpd at 4 Hz for chromatic and achromatic gratings shown within 11.9 × 7.8 degrees of visual field. They found a low-pass spatial tuning function for chromatic stimuli and a band-pass function for the achromatic case. Arakawa et al. 19 found a steeper decrement of the chromatic response at high spatial frequencies compared to our results. This difference might be explained by the fact that they measured sensitivity using suprathreshold amplitudes. It is interesting to note that, for chromatic evoked potentials, there appears to be a disagreement between transient and steady-state stimulation at the amplitude-contrast functions measured at suprathreshold contrasts. For instance, transient chromatic VECP amplitude has a band-pass tuning function with sharp attenuation at the higher and lower spatial frequencies 23.

At 8 cpd, the sensitivity was a bit higher than at 2.4 and 6 cpd. This discrepancy is probably related to a low SNR value at that frequency. Because the selection of data points to be measured was based on the Meigen and Bach method 33, this caused the amount of data points to be measured in each subject to be less than at the other frequencies, which makes the regression lines extrapolate to zero amplitude in lower contrasts than if all the amplitude values were considered for measurement.

The fact that psychophysical and evoked potential thresholds were similar when using the same temporal frequency is in agreement not only with previous studies that compared VECP and behavioral data but also with studies about the tuning of the psychophysical chromatic contrast sensitivity function 11. The low-pass tuning for red-green sinusoidal gratings has been well described by Mullen 11. Despite the fact that the static psychophysical thresholds were higher than VECP and dynamic psychophysical thresholds, the low-pass characteristic of the static psychophysical function was maintained. Psychophysical thresholds estimated with static stimulation were significantly higher than electrophysiological or dynamic psychophysical thresholds. The difference between static and dynamic psychophysical results could be understood on the basis of chromatic adaptation caused by the threshold estimation used. For static presentation, when decreasing the contrast in order to find the threshold, the subjects did report what seems to be an after-image effect similar to the effect reported when changing contrast abruptly from its maximum value to the background of mean chromaticity and luminance. In the dynamic psychophysics protocol the reversal of the gratings minimizes this effect, thus providing higher thresholds. It is interesting to note that the ssVECP thresholds did not receive a contribution from this effect, probably because they occurred in very low contrast.

### Evoked potentials and psychophysics for blue-yellow gratings

The ssVECP amplitudes were lower for blue-yellow compared to red-green gratings. This decrease in amplitude has been reported since Regan's study in 1973 21 and can be attributed to koniocellular pathway properties such as fewer projections from retina to LGN and lack of S cones in the central fovea.

The VECP function for blue-yellow stimulation had no spatial tuning and was different for both psychophysical conditions, which were low-pass tuned. This happened mainly because ssVECP thresholds were lower at the higher spatial frequencies in comparison to psychophysics. It is possible that a luminance intrusion could have caused this difference. Some precautions were taken to avoid chromatic aberration when stimulating with blue-yellow gratings. First, all subjects performed the Heterochromatic Flicker Photometry task before each test. Second, the size of the field was small (5°). However, chromatic aberration cannot be ruled out. Kulikowski and Robson 39 used transient on-off VECPs elicited by stimulation with blue-yellow gratings at 2 cpd with 3° and 6° field size. For the latter, VECP showed degraded responses beyond 4 cpd. Our results show something similar in Figure 3B, where it can be seen that a major difference between VECP and both psychophysical tests occurs from 4 to 8 cpd.

If this difference cannot be fully attributed to chromatic aberration in view of our precautions, it might perhaps be related to the macular pigment (MP). Robson et al. 40 evaluated subjects with either transient or steady-state equiluminant VECP. They used gratings of various field sizes and quantified the achromatic response intrusion by the onset of reversal-like waves in onset/offset transient VECP waveform or by the decreased amplitude in fundamental harmonics for ssVECP. Their results clearly showed a minor influence of chromatic aberration in subjects with less MP. Subjects with dense MP showed VECP with waveform and fundamental frequency indicative of luminance intrusion. It was suggested that MP could be important to generate luminance intrusion, which should be weighted with the chromatic aberration influence.

We conclude that, despite the shallower decrease at the high spatial frequencies observed in our results for red-green chromatic sensitivity, VECP sensitivities were similar to those measured for suprathreshold amplitude as well as to CSF measured by threshold estimates reported in other studies. In contrast to suprathreshold measurements, CSFs determined at a range of contrast levels can provide a better estimate of chromatic discrimination in spite of the longer testing time and the more laborious procedure. CSFs measured with ssVECP for red-green gratings can be reliably used to evaluate chromatic CSFs in agreement with psychophysics, especially for stimuli with similar temporal properties. Care must be taken when using spatial frequencies 4 cpd or higher to elicit blue-yellow VECPs since chromatic aberration might quickly degrade the response, impairing a comparison with psychophysics. What is new in our study is the use of a threshold estimate taken from a range of contrasts for both red-green and blue-yellow stimuli and the use of pooled cone contrast metrics. In addition, the present study corroborates previous research showing that suprathreshold measurements obtained by ssVECP correlate well with psychophysical procedures for red-green CSF and draws attention to the overestimated thresholds provided by ssVECP at spatial frequencies 4 cpd or higher in comparison with psychophysical thresholds for blue-yellow CSF.
